# Validation and reliability test of Chinese language patient-reported impact of symptoms in schizophrenia scale

**DOI:** 10.3389/fpsyt.2023.1158937

**Published:** 2023-05-24

**Authors:** Xiao Lin, Hongjun Tian, Lina Wang, Ranli Li, Xiaoyan Ma, Yun Sun, Ziyao Cai, Jing Ping, Langlang Chen, Chuanjun Zhuo, Haiping Yu

**Affiliations:** ^1^Department of Psychiatry, Wenzhou Seventh Peoples Hospital, Wenzhou, China; ^2^Key Laboratory of Sensor Information Processing Abnormalities in Schizophrenia (SIPAS-Lab), Tianjin Fourth Center Hospital, Nankai University Affiliated Tianjin Fourth Center Hospital, Tianjin, China; ^3^Key Laboratory of Sensor Information Processing Abnormalities in Schizophrenia (SIPAS-Lab), Tianjin Fourth Center Hospital, Tianjin Medical University Affiliated Tianjin Fourth Center Hospital, Tianjin, China; ^4^Laboratory of Psychiatric-Neuroimaging-Genetics and Co-morbidity, Tianjin Anding Hospital, Tianjin Mental Health Center of Tianjin Medical University, Tianjin, China; ^5^Department of Psychiatry, Tianjin Anding Hospital, Tianjin, China

**Keywords:** validity, reliability, schizophrenia, CL-PRISS, CFA

## Abstract

**Background:**

Patient-reported outcomes, or subjective evaluations directly reflecting the patient’s views, feelings, and judgments, are now being used to evaluate the outcomes of care and treatment of people with schizophrenia. In this study, we used an updated tool, the patient-reported impact of symptoms in schizophrenia scale (PRISS), translated into Chinese languages to assess the subjective experiences of schizophrenia patients.

**Objective:**

This study aimed to test the psychometrics of the Chinese languages PRISS (CL-PRISS).

**Method:**

This study used the Chinese version of PRISS (CL-PRISS), acquired from the harmonized English-language version. A total of 280 patients enrolled in this study were asked to complete the CL-PRISS, the positive and negative syndrome scale (PANSS), and the World Health Organization Disability Assessment Schedule (WHO-DAS). Construct and concurrent validity was tested using the confirmatory factor analysis (CFA) and Spearman correlation coefficient, respectively. The reliability of CL-PRISS was tested using Cronbach’s α coefficient and the internal correlation coefficient.

**Results:**

Confirmatory factor analysis (CFA) analysis demonstrated three major factors in CL_PRISS: the first factor is productive experiences, the second factor is affective-negative, and the third factor experiences. The factor loadings between items and factors ranged from 0.436 to 0.899 (RMSEA = 0.029, TLI = 0.940, CFI = 0.921). The correlation coefficient between the CL_PRISS and PANSS was 0.845, and between the CL-PRISS and WHO-DAS was 0.886. The ICC of the total CL_PRISS was 0.913, and Cronbach’s α was 0.903.

**Conclusion:**

The Chinese version of the PRISS (CL_PRISS) can be effectively used for assessing the subjective experience of Chinese patients with schizophrenia.

## Introduction

Schizophrenia is a chronic disabling disease, usually accompanied by delusion, hallucination, poverty of thought, apathy, avolition, blunted affect, behavioral disorganization, and cognitive impairments (1–3). More than 60% of patients with schizophrenia do not acquire long-term remission. Hence, this disease requires long-term management to reduce side effects and improve the long-term treatment effect (4–6). Patients with schizophrenia are monitored at regular intervals using multiple assessment tools to check the effect of the ongoing treatment and changes in care management (7–9). Each of the tools used to assess the symptoms of schizophrenia patients has priority and assesses only a hand full of symptoms at a time. Among these tools, the positive and negative syndrome scale (PANSS) is considered the gold standard. It is used to assess the core symptoms of schizophrenia (10). The other frequently used tools are the brief psychiatric rating scale (BPRS) (11), the psychopathology rating schedule (PRS) (12), the scale for the assessment of negative symptoms (SANS) (13), and the scale for the assessment of positive symptoms (SAPS) (14). Though these frequently used tools can assess patients with schizophrenia from multiple or specific dimensions, they are all observer rating scales. These tools do not provide information on patients’ subjective experiences with schizophrenia.

Accompanied by the progress of world health management, schizophrenia long-term management also needed advancement (15–19). Based on this background and the patients’ experience, management (including antipsychotic, lifestyle, and rehabilitation managements) was proposed (15, 20, 21). Back on this ground, patient-based healthy management was developed in the last two decades in the clinical practices of patients with mental disorders (22, 23). To the best of our knowledge, only about 16.6% of patients with schizophrenia can achieve recovery after accepting antipsychotic treatment (24). Due to the lower recovery of patients with schizophrenia, long-term regular evaluation and treatment should be administrated by tailored antipsychotic agent treatment in clinical practices (25). Tailored treatment is needed to precisely evaluate patients with schizophrenia from multiple dimensions (25, 26). Because patients with schizophrenia need regular evaluation, assessment tools should be feasible for clinical use (27).

Involving patients in their healthcare and assessing the benefits of treatment from the patient’s perspective is becoming increasingly popular and is thought to be useful in the prognosis of many diseases and disorders. The Food and Drug Administration (FDA) advocates the use of patient-reported outcomes and defines it as “any report of the status of a patient’s health condition that comes directly from the patient without interpretation of the patient’s response by a clinician or anyone else” (28). Studies have shown that patient-rated outcome from schizophrenia patients provides a better guide to the quality of care and shows consistent positive associations between patient-reported experience and clinical effectiveness.

There are many patient-reported outcome measures (PROMs) designed to assess patients with schizophrenia. Each of these measures has different conceptual perspectives that can be condition-specific, such as the schizophrenia quality of life scale, or generic, like Warwick-Edinburgh mental wellbeing scale, which applies to more than one mental health condition. The three-level EuroQoL five-dimension (EQ-5D) and the short-form six-dimension (SF-6D) are used for preference-based measures. Miller and Chouinard (12), in their review, identified 70 PROMs used to evaluate patients with schizophrenia. These tools are categorized into six domains that focus on health-related quality of life, insight, depression/feelings, treatment-related illness symptoms, or caregiver/family. Some questionnaires also relate to personality measurement, communication between patients and clinicians, and service satisfaction. However, the authors concluded that none of these tools used singularly was sufficient to give a clear understanding of the condition and treatment effects in schizophrenia patients, and there is a need for developing new PROMs tools.

In this study, we tested the patient-reported impact of symptoms on schizophrenia scale (PRISS) developed by Moreno-Küstner et al. (29) to assess the subjective impact of reported experiences related to the main symptoms in patients with schizophrenia. The structural and convergent validity, test-retest reliability, and internal consistency of this tool were conducted in outpatient and community rehabilitation settings in Spain (29). This tool is ideal for clinical use and can be used on patients with schizophrenia requiring long-term treatment. To use this tool on Chinese patients, we translated the PRISS into a Chinese language version. Here, we report the validity and reliability of the Chinese language PRISS (CL-PRISS) in schizophrenia patients.

## Materials and methods

### Participants

All subjects were recruited from the Department of Psychiatry of Tianjin Anding Hospital, the Department of Psychiatry of Tianjin Fourth Center Hospital, and the Department of Psychiatry of Wenzhou Seventh Peoples’ Hospital from September 2021 to October 2022. The inclusion criteria were: (1) ≥ 18 years; (2) diagnosed with schizophrenia by a professional psychiatrist according to the criteria of DSM-IV; (3) the patients had insight; (4) the patients’ symptoms were stable; (5) could understand the CL-PRISS, (6) could self-report their subjective experiences clearly; (7) without intellectual disability; (8) without neurodegenerative disease; (9) without a history of personality disorder; (10) without substance abuse, except mild nicotine dependence according to the Fagerstrom test for nicotine dependence (FTND) (30); (11) without brain trauma; and (12) without any other factors which possibly interfere with the subject insights. The exclusion criteria were: (1) moderate and severe nicotine dependences; (2) having a history of other mental disorders, such as combined with depression; (3) having IQ < 80; (4) with a history of epilepsy; and (5) with a history of severe physical disease. The Ethics Committee of Tianjin fourth Center Hospital approved this study (IRB No. TW- 2022-08-22). All the guardians of the subjects provided signed informed consent.

### PRISS introduction

The PRISS has 28 items. They are as follows: (1) delusions; (2) conceptual disorganization; (3) hallucinations; (4) excitement; (5) grandiosity; (6) suspiciousness; (7) hostility; (8) blunted affect; (9) poor rapport; (10) passive social withdrawal; (11) difficulty in abstract thinking; (12) lack of spontaneity; (13) stereotyped thinking; (14) somatic concern; (15) anxiety; (16) feelings of guilt; (17) tension; (18) mannerisms; (19) depression; (20) motor retardation; (21) uncooperativeness; (22) unusual thought content; (23) disorientation; (24) poor attention; (25) disturbance of volition; (26) poor impulse; (27) preoccupation; and (28) active social avoidance. The four characteristics of PRISS (presence, frequency, worry, and interference with daily life) are recorded for each item. The PRISS is a rater scale. The experience level of subjects is assessed with a Likert scale. For example, the frequency of any experience is recorded on a 4-point Likert scale using 1: almost never; 2: sometimes; 3: often; or 4: always. The level of concern (worry) and its interference in the daily life of the patient is scored on a 5-point Likert scale from 0 to 4, where 0: no concern/no interference; 1: mild/slight; 2: moderate; 3: serious; and 4: extreme.

### Localization and optimization

We translated the PRISS into the Chinese language and then back-translated the Chinese languages (CL-PRISS) to English-language versions with the help of a native English-language speaker (S. Patricia Chou). The final version of CL-PRISS was acquired from the harmonized English-language version. The CL-PRISS was used to assess the subjective impact of PRISS items on schizophrenia patients.

### Validity evaluation

Confirmatory factor analysis (CFA) was used to determine structural validity, and the variance maximum method was used to calculate the factors and factor loads (30, 31). Spearman rank correlation coefficients were used to determine concurrent validity. PANSS and World Health Organization Disability Assessment Schedule (WHO-DAS) were adapted to the criterion (32).

### Reliability evaluation

All the patients were assessed independently by 12 raters. The raters knew patient diagnoses but were blinded to each other’s scores. Internal correlations (ICCs) were used to assess the inter-consistency. Cronbach α coefficient of the full sample was used to determine split-half reliability (33).

### Definition of the cut-off point

The clinical standard provided by consensus of 12 professional doctors treating schizophrenia for more than 15 years was taken as a reference in this study. The area under the receiver operating characteristic curve (AUC) (34), acceptable to the subject, judged the cut-off points for the severity of self-reported suffering of symptoms of schizophrenia. Cut-off points and their sensitivity and specificity are all calculated for quantitative assessment of suffering severity.

### Statistical analysis

SPSS 21.0 (SPSS Inc., Chicago, IL, USA) was used for variables statistical analyses. The relationships between CL-PRISS and PANSS/WHO-DAS scores were analyzed by the Pearson/Spearman correlation test. The internal consistency of the scale was evaluated by calculating Cronbach’s α coefficient and ICC. In this study, the explore factors analysis (EFA) was used to test whether the scale can be used to conduct CFA analysis or not. The factor loading was found to be above 0.4, the adequacy of Kaiser–Meyer–Olkin (KMO) above 0.900 was used as the reference to assess the PRISS can be suitable to conduct CFA analysis or not. Confirm factors analysis (CFA) was conducted with a weighted least squares, mean, and variance adjusted estimator that enables treatment of ordinal data in Mplus, version 7.4 (35, 36). In the CFA, model fitness was determined based on the comparative fit index (CFI), Tucker–Lewis index (TLI), and root-mean-square error of approximation (RMSEA) value; all three of these indices are well established as effective and reliable indicators (35, 36). The criteria for an acceptable model fit were: CFI > 0.90, TLI > 0.90, and RMSEA < 0.08 (35–38). As an additional measure of model fitness, we calculated the quotient of the minimum discrepancy, Ĉ, and degrees of freedom (DF), written Cmin/DF. A | Cmin/DF value| < 3 indicated an acceptable fit. To investigate a hypothesized PRISS structure, a theoretical 1-factor (mental suffering) structure was imposed while treating age and gender as covariates.

## Results

### Socio-demographic and clinical information of the patients

This study included 160 female and 120 male patients with schizophrenia. The age of the patients ranged between 18 and 45 years of age (Mean age = 31.30, SD = 5.20). The educational level varied from 3 to 18 years (Mean years = 15.25, SD = 5.22). The mean illness duration was 1∼10 years (Mean years = 6.56, SD = 3.20). The mean daily dosage of antipsychotics ranged from 450∼1,025 mg (Chlorpromazine equivalent; mean daily dosage = 680.50.50, SD = 200.00). There was no significant difference between female and male patients regarding age, educational level, illness duration, and the daily dosage of antipsychotic agents.

### Construct validity

The data of explore elemental psychopathy assessment (EPA) demonstrated that two factors of the PRISS were labeled as follows: presence-frequency (12 items), worry about the symptoms, life quality influence (6 items) and life quality influence (10 items). Simultaneously, all the factor loading was found to be above 0.4. The data of EFA was performed on scores from a randomly selected subsample (*n* = 280). The adequacy of KMO (0.900) and significance of Bartlett’s test of sphericity (χ2 = 1,036.546, *P* < 0.001 and χ2 = 1,469.285, *P* < 0.001, respectively) verified the appropriateness of the sample for factor analysis. PRISS assesses three dimensions of the patients: presence-frequency, worry about the symptoms, and interference with daily life. In each dimension, the sample data were suitable for factor analysis based on the KMO measure and Bartlett’s test of sphericity (39). In this study, the KMO of 0.932 and Bartlett’s χ2 value of 3,028.789 (*P* < 0.01) met the conditions for CFA. CFA demonstrated three factors in the 28 items, and the contribution rate of the three factors was 72.400% (the first dimension: RMSEA = 0.02190, TLI = 0.955, CFI = 0.969; the second dimension, RMSEA = 0.045, TLI = 0.964, CFI = 0.952; the third dimension: RMSEA = 0.042, TLI = 0.972, CFI = 0.969). The factor loading of each dimension of the factor is listed in Tables 1–9. The correlation between the factors is listed in Tables 1–9.

**TABLE 1:** CL-PRISS’ first dimensions item’s factor loading in the first factor.

CL-PRISS items’ factor loading in the first factor (gained from CFA)		
**Items**	**Items describe**	**Factor loading**
1	Delusions	0.581, 0.477∼0.800
3	Hallucinations	0.665, 0.500∼0.855
5	Grandiosity	0.720, 0.575∼0.824
6	Suspiciousness	0.829, 0.623∼0.954
13	Stereotyped thinking	0.822, 0.611∼0.900
18	Mannerisms and posturing	0.620, 0.528∼0.865
21	Uncooperativeness	0.700, 0.646∼0.900
22	Unusual thought content	0.830, 0.741∼0.928
23	Disorientation	0.600, 0.522∼0.805
26	Poor impulse control	0.545, 0.400∼0.697

The factor defined as productive experience.

**TABLE 2:** CL-PRISS’ first dimensions item’s factor loading in the second factor.

CL-PRISS items’ factor loading in the second factor (gained from CFA)		
**Items**	**Items describe**	**Factor loading**
2	Conceptual disorganization	0.530, 0.466∼0.662
8	Blunted affect	0.650, 0.554∼0.721
9	Poor rapport	0.633, 0.500∼0.722
10	Passive social withdrawal	0.725, 0.602∼0.833
11	Difficulty in abstract thinking	0.623, 0.506∼0.883
12	Lack of spontaneity	0.785, 0.599∼0.966
15	Anxiety	0.830, 0.645∼0.899
16	Feelings of guilt	0.700, 0.634∼0.795
19	Depression	0.754, 0.533∼0.784
20	Motor retardation	0.625, 0.477∼0.639
24	Poor attention	0.850, 0.690∼0.900
25	Disturbance of volition	0.674, 0.458∼0.823
27	Preoccupation	0.524, 0.433∼0.690
28	Active social avoidance	0.652, 0.544∼0.745

The factor defined as: affective-negative.

**TABLE 3:** CL-PRISS’ first dimension item’s factor loading in the third factor.

CL-PRISS items’ factor loading in the third factor (gained from CFA)		
**Items**	**Items describe**	**Factor loading**
4	Excitement	0.670, 0.593∼0.872
7	Hostility	0.840, 0.692∼0.998
14	Somatic concern	0.680, 0.667∼0.887
17	Tension	0.704, 0.568∼0.925

The factor defined as excitation.

**TABLE 4:** CL-PRISS’ second dimension item’s factor loading in the first factor.

CL-PRISS items’ factor loading in the second factor (gained from CFA)		
**Items**	**Items describe**	**Factor loading**
1	Delusions	0.730, 0.664∼0.889
3	Hallucinations	0.750, 0.590∼0.924
5	Grandiosity	0.833, 0.758∼0.965
13	Stereotyped thinking	0.720, 0.596∼0.941
18	Mannerisms and posturing	0.787, 0.633∼0.978
21	Uncooperativeness	0.821, 0.698∼0.989
22	Unusual thought content	0.822, 0.738∼0.985
26	Poor impulse control	0.727, 0.555∼0.833

The factor defined as productive experience.

**TABLE 5:** CL-PRISS’ second dimension item’s factor loading in the second factor.

CL-PRISS items’ factor loading in the second factor (gained from CFA)		
**Items**	**Items describe**	**Factor loading**
2	Conceptual disorganization	0.617, 0.522∼0.740
6	Suspiciousness	0.852, 0.620∼0.844
8	Blunted affect	0.644, 0.566∼0.785
9	Poor rapport	0.652, 0.590∼0.788
10	Passive social withdrawal	0.752, 0.658∼0.887
11	Difficulty in abstract thinking	0.600, 0.534∼0.825
12	Lack of spontaneity	0.635, 0.500∼0.775
15	Anxiety	0.639, 0.544∼0.737
16	Feelings of guilt	0.725, 0.600∼0.903
19	Depression	0.647, 0.558∼0.817
20	Motor retardation	0.678, 0.603∼0.781
23	Disorientation	0.842, 0.728∼0.909
24	Poor attention	0.625, 0.575∼0.814
25	Disturbance of volition	0.693, 0.600∼0.756
27	Preoccupation	0.610, 0.599∼0.747
28	Active social avoidance	0.654, 0.603∼0.711
17	Tension	0.533, 0.475∼0.735

The factor defined as affective- negative.

**TABLE 6:** CL-PRISS’ second dimension item’s factor loading in the third factor.

CL-PRISS items’ factor loading in the third factor (gained from CFA)		
**Items**	**Items describe**	**Factor loading**
4	Excitement	0.835, 0.693∼0.927
7	Hostility	0.655, 0.509∼0.747
14	Somatic concern	0.478, 0.359∼0.687

The factor defined as: excitation.

**TABLE 7:** CL-PRISS’ second dimension item’s factor loading in the first factor.

CL-PRISS items’ factor loading in the second factor (gained from CFA)		
**Items**	**Items describe**	**Factor loading**
1	Delusions	0.730, 0.664∼0.889
3	Hallucinations	0.750, 0.590∼0.924
5	Grandiosity	0.833, 0.758∼0.965
13	Stereotyped thinking	0.720, 0.596∼0.941
18	Mannerisms and posturing	0.787, 0.633∼0.978
21	Uncooperativeness	0.821, 0.698∼0.989
22	Unusual thought content	0.822, 0.738∼0.985
26	Poor impulse control	0.727, 0.555∼0.833

The factor defined as productive experience.

**TABLE 8:** CL-PRISS’ second dimension item’s factor loading in the second factor.

CL-PRISS items’ factor loading in the second factor (gained from CFA)		
**Items**	**Items describe**	**Factor loading**
2	Conceptual disorganization	0.825, 0.750∼0.930
6	Suspiciousness	0.744, 0.547∼0.875
8	Blunted affect	0.669, 0.600∼0.828
9	Poor rapport	0.745, 0.702∼0.902
10	Passive social withdrawal	0.647, 0.543∼0.852
11	Difficulty in abstract thinking	0.653, 0.525∼0.773
12	Lack of spontaneity	0.693, 0.558∼0.735
15	Anxiety	0.730, 0.640∼0.923
16	Feelings of guilt	0.689, 0.558∼0.825
19	Depression	0.666, 0.593∼0.784
20	Motor retardation	0.525, 0.470∼0.714
24	Poor attention	0.644, 0.596∼0.765
25	Disturbance of volition	0.617, 0.600∼0.733
27	Preoccupation	0.554, 0.503∼0.598
28	Active social avoidance	0.831, 0.731∼0.0949

The factor defined as: affective- negative.

**TABLE 9:** CL-PRISS’ third dimension item’s factor loading in the third factor.

CL-PRISS items’ factor loading in the third factor (gained from CFA)		
**Items**	**Items describe**	**Factor loading**
18	Mannerisms and posturing	0.885, 0.784∼0.930
22	Unusual thought content	0.625, 0.500∼0.732
26	Poor impulse control	0.678, 0.559∼0.882
15	Anxiety	0.882, 0.668∼0.989
16	Feelings of guilt	0.834, 0.777∼0.911
19	Depression	0.589, 0.477∼0.723
27	Preoccupation	0.667, 0.559∼0.884
4	Excitement	0.812, 0.748∼0.903
7	Hostility	0.699, 0.588∼0.777
14	Somatic concern	0.825, 0.759∼0.880
17	Tension	0.790, 0.643∼0.830

The factor defined as excitation.

#### Concurrent validity

Our analysis showed that the Spearman rank correlation coefficient was 0.920 between CL-PRISS and the PANSS and 0.850 between CL-PRISS and WHO-DAS.

### Reliability data

#### Inter-rater consistency

The total CL-PRISS’ ICC value was 0.852. The Cronbach α coefficient of the total CL_PRISS was 0.848 showing good reliability.

### Receiver operating characteristic (ROC) discriminated cut-off point

Taking the clinical evaluation standard of personality features as a reference, ROC demonstrated that the cut-off score was ≥ 33. The sensitivity was 0.968, the specificity was 0.882, and the area under the curve (AUC) was 0.798. Compared to the clinical definition, the cut-off point was 33. The definition provided a severe symptom impact of schizophrenia. The cut-off score was ≥ 22, accompanied by a sensitivity of 0.917 and specificity of 0.855, and the AUC was 0.773. Compared to the clinical definition, the cut-off point was 22; the definition provided a moderate symptom impact of schizophrenia. The cut-off score was ≥ 12, accompanied by a sensitivity of 0.908 and specificity of 0.833, and the AUC was 0.798. Compared to the clinical definition, the cut-off point was 12; the definition provided a mild symptom impact of schizophrenia.

## Discussion

The data provided in this study confirms that CL-PRISS has ideal psychometric measures that can be used to examine the subjective experiences of patients with schizophrenia (Table 10). The validity and reliability of CL-PRISS are higher than other psychometric standards. Further, the ROC analysis demonstrated that CL-PRISS can be used to evaluate the severity of the impact induced by the subjective experiences of the symptoms. Subjective suffering plays a pivotal role in patient-based treatment strategy making. This tool will help us understand the subjective suffering induced by the symptoms from the patient’s perspective. In addition, from the perspective of clinical physicians, CL-PRISS, as a patient-reported tool, had the same effectiveness in the long-term management of patients with schizophrenia as the patient-based long-term management plan.

**TABLE 10:** Chinese version of PRISS.

中文版精神分裂症症状影响量表患者自评量表	
条目	
1.	妄想
2.	思维紊乱
3.	幻觉
4.	激越
5.	夸大
6.	多疑
7.	敌意
8.	情感平淡
9.	交流障碍
10.	被动交流，社会退缩
11.	抽象思维困难
12.	缺乏自发性
13.	刻板思维
14.	躯体感觉不适
15.	焦虑
16.	负罪感
17.	紧张
18.	行为幼稚
19.	抑郁
20.	行动迟缓
21.	不合作
22.	思维内容异常
23.	定向力紊乱
24.	注意力下降
25.	意志活动紊乱
26.	缺乏主动性
27.	先占观念
28.	主动回避社交

Validity plays a pivotal role in defining any scale. It can provide precise information for clinical practices. The construct validity of CL-PRISS was confirmed by CFA. The KMO of 0.889, Bartlett’s χ2 value of 3,028.789 (*P* < 0.01), and the cumulative variance contribution rate of 72.400% indicate that the constructive validity of CL-PRISS is ideal and can be used as an assessment scale. The Spearman rank correlation coefficient was 0.923 between CL-PRISS and the PANSS and 0.917 between CL-PRISS and WHO-DAS. This demonstrates that CL_PRISS has ideally concurrent validity.

Reliability is very important for the raters to use a scale to assess the patient with schizophrenia. Good reliability provides consistent information from the screening of patients among different raters. Our data demonstrated that the inter-rater consistency gained from the internal correlation coefficient and split-half reliability gained from Cronbach α coefficient analysis all converged to support that CL-PRISS was ideally reliable. Notably, using the ROC method, our data demonstrated that the scores of CL-PRISS could be used to discriminate the mild, moderate, and severe subjective sufferings induced by the symptoms of the patients with schizophrenia.

We have shown that CL-PRISS has three dimensions, and each dimension has three factors. The first factor is productive experiences, the second is affective-negative, and the third is excitation. The factor loading of all 28 items in these three factors was above 0.400. This supports the notion that the three dimensions of PRISS have good constructive validity and the assessment can cover the six factors of CL-PANSS (in the PANSS, the first factor is positive, the second factor is negative, the third factor is activation, the fourth factor is an effect, the fifth factor is disorganization, and the sixth factors is resistance) (40, 41). CL-PANSS is an ideal tool for clinical doctors to assess the severity of illness in patients with schizophrenia. CL-PRISS is an ideal tool for schizophrenia patients to report their subjective experience (suffering) induced by the symptoms. Hence, for optimal long-term management of patients with schizophrenia, CL-PRISS and CL-PANSS play pivotal roles in pursuing a better prognosis (42–45).

## Limitation

There are three limitations in the present study. First, although CL-PRISS has a highlighted correlation with PANSS, it cannot replace it, as CL-PANSS is inclined to address the self-suffering of the patients. Secondly, CL-PRISS can acquire a global assessment of the self-suffering of Chinese patients with schizophrenia if the clinical doctor aims to describe the unusual symptoms of the patient’s suffering. In this respect, other tools may be more suitable. For example, if the doctor wants to address auditory verbal hallucinations, AVH-scale is more suitable than CL-PRISS. The third limitation is that although the CL-PRISS can discriminate mild, moderate, and severe self-suffering symptoms of schizophrenia, it should be re-tested in a large sample cohort study for further clarification (42–45).

## Conclusion

Our data demonstrated that CL-PRISS has ideal validity and reliability and can be used to routinely monitor the symptoms’ impact on Chinese patients with schizophrenia.

## Data availability statement

The raw data supporting the conclusions of this article will be made available by the authors, without undue reservation.

## Ethics statement

The studies involving human participants were reviewed and approved by the Ethics Committee of Tianjin Fourth Center Hospital. The patients/participants provided their written informed consent to participate in this study.

## Author contributions

XL, HT, LW, RL, XM, and YS: conceptualization, methodology, software, analysis, investigation, and writing/original draft preparation. ZC, JP, LC, HY, and CZ: software, analysis, writing/reviewing, and editing. XL, CZ, and HT: conceptualization and supervision. All authors read and approved the final version of the manuscript.

## References

[1] Abplanalp S Braff D Light G Nuechterlein K Green M. Understanding connections and boundaries between positive symptoms, negative symptoms, and role functioning among individuals with schizophrenia: a network psychometric approach. *JAMA Psychiatry.* (2022) 79:1014–22. 10.1001/jamapsychiatry.2022.2386 35976655 PMC9386606

[2] Ahmed A Kirkpatrick B Granholm E Rowland L Barker P Gold J Two factors, five factors, or both? external validation studies of negative symptom dimensions in schizophrenia. *Schizophr Bull.* (2022) 48:620–30. 10.1093/schbul/sbab148 35020936 PMC9077418

[3] Wang L Lam C Huang J Cheung E Lui S Chan R. Range-adaptive value representation in different stages of schizophrenia: a proof of concept study. *Schizophr Bull.* (2021) 47:1524–33. 10.1093/schbul/sbab099 34420057 PMC8530390

[4] Lopez-Morinigo J Leucht S Arango C. Pharmacological treatment of early-onset schizophrenia: a critical review, evidence-based clinical guidance and unmet needs. *Pharmacopsychiatry.* (2022) 55:233–45. 10.1055/a-1854-0185 35777418 PMC9458343

[5] Ostuzzi G Vita G Bertolini F Tedeschi F De Luca B Gastaldon C Continuing, reducing, switching, or stopping antipsychotics in individuals with schizophrenia-spectrum disorders who are clinically stable: a systematic review and network meta-analysis. *Lancet Psychiatry.* (2022) 9:614–24. 10.1016/s2215-0366(22)00158-4 35753323

[6] Spark D Fornito A Langmead C Stewart G. Beyond antipsychotics: a twenty-first century update for preclinical development of schizophrenia therapeutics. *Transl Psychiatry.* (2022) 12:147. 10.1038/s41398-022-01904-2 35393394 PMC8991275

[7] Nibbio G Barlati S Calzavara-Pinton I Necchini N Invernizzi E Dell’Ovo D Assessment and correlates of autistic symptoms in Schizophrenia Spectrum Disorders measured with the PANSS Autism Severity Score: a systematic review. *Front Psychiatry.* (2022) 13:934005. 10.3389/fpsyt.2022.934005 36111306 PMC9468543

[8] Ince Guliyev E Guloksuz S Ucok A. Impaired effort allocation in patients with recent-onset schizophrenia and its relevance to negative symptoms assessments and persistent negative symptoms. *J Clin Med.* (2022) 11:5060. 10.3390/jcm11175060 36078990 PMC9457458

[9] Burgin S Reniers R Humpston C. Prevalence and assessment of self-disorders in the schizophrenia spectrum: a systematic review and meta-analysis. *Sci Rep.* (2022) 12:1165. 10.1038/s41598-022-05232-9 35064201 PMC8782935

[10] Kay S Fiszbein A Opler L. The positive and negative syndrome scale (PANSS) for schizophrenia. *Schizophr Bull.* (1987) 13:261–76. 10.1093/schbul/13.2.261 3616518

[11] Overall J Gorham D. The brief psychiatric rating scale. *Psychol Rep.* (1962) 10:799–812. 10.2466/pr0.1962.10.3.799

[12] Miller R Chouinard G. The psychosis rating scale (PRS): a new scale for positive symptoms of psychosis derived from neurodynamic theory. *Schizophr Res.* (1998) 29:37–41. 10.1016/S0920-9964(97)88382-X

[13] Andreasen N. The Scale for the Assessment of Negative Symptoms (SANS): conceptual and theoretical foundations. *Br J Psychiatry Suppl.* (1989):155:49–58. 2695141

[14] Andreasen N. *Scale for the Assessment of Positive Symptoms (SAPS).* Iowa City, IA: University of Iowa (1984).

[15] Carswell C Brown J Lister J Ajjan R Alderson S Balogun-Katung A The lived experience of severe mental illness and long-term conditions: a qualitative exploration of service user, carer, and healthcare professional perspectives on self-managing co-existing mental and physical conditions. *BMC Psychiatry.* (2022) 22:479. 10.1186/s12888-022-04117-5 35850709 PMC9295434

[16] Peng M Ma Z Ran M. Family caregiving and chronic illness management in schizophrenia: positive and negative aspects of caregiving. *BMC Psychol.* (2022) 10:83. 10.1186/s40359-022-00794-9 35361263 PMC8973811

[17] Citrome L Belcher E Stacy S Suett M Mychaskiw M Salinas G. Management of schizophrenia with long-acting injectable antipsychotic medications: an assessment of the educational needs of clinicians. *Neuropsychiatr Dis Treat.* (2022) 18:111–23. 10.2147/ndt.s326299 35115779 PMC8801366

[18] Rancans E Dombi Z Barabássy Á. Dosing cariprazine within and beyond clinical trials: recommendations for the treatment of schizophrenia. *Front Psychiatry.* (2021) 12:770234. 10.3389/fpsyt.2021.770234 35069278 PMC8768837

[19] Lavaud P McMahon K Sánchez Rico M Hanon C Alvarado J de Raykeer R Long-term care utilization within older adults with schizophrenia: associated factors in a multicenter study. *Psychiatry Res.* (2022) 308:114339. 10.1016/j.psychres.2021.114339 34963089

[20] Aimola L Gordon-Brown J Etherington A Zalewska K Cooper S Crawford M. Patient-reported experience and quality of care for people with schizophrenia. *BMC Psychiatry.* (2019) 19:17. 10.1186/s12888-018-1998-y 30626355 PMC6327578

[21] van Dam M van Weeghel J Stiekema A Castelein S Pijnenborg M van der Meer L. Barriers and facilitators to implementation of cognitive adaptation training in long-term inpatient facilities for people diagnosed with severe mental illness: a nursing perspective. *J Psychiatr Ment Health Nurs.* (2022) 29:568–77. 10.1111/jpm.12821 35048468 PMC9543523

[22] Comulada W Swendeman D Koussa M Mindry D Medich M Estrin D Adherence to self-monitoring healthy lifestyle behaviours through mobile phone-based ecological momentary assessments and photographic food records over 6 months in mostly ethnic minority mothers. *Public Health Nutr.* (2018) 21:679–88. 10.1017/s1368980017003044 29199630 PMC5807077

[23] Anwar N Kuppili P Balhara Y. Depression and physical noncommunicable diseases: the need for an integrated approach. *WHO South East Asia J Public Health.* (2017) 6:12–7. 10.4103/2224-3151.206158 28597853

[24] Toll A Blanco-Hinojo L Bergé D Duran X Canosa I Legido T Multidimensional predictors of negative symptoms in antipsychotic-naive first-episode psychosis. *J Psychiatry Neurosci.* (2022) 47:E21–31. 10.1503/jpn.210138 35046133 PMC8789336

[25] Gowda G Isaac M. Models of care of schizophrenia in the community-an international perspective. *Curr Psychiatry Rep.* (2022) 24:195–202. 10.1007/s11920-022-01329-0 35230610 PMC8967793

[26] Rodrigues J Martinho A Santa C Madeira N Coroa M Santos V Systematic review and meta-analysis of mass spectrometry proteomics applied to human peripheral fluids to assess potential biomarkers of schizophrenia. *Int J Mol Sci.* (2022) 23:4917. 10.3390/ijms23094917 35563307 PMC9105255

[27] Salvador-Carulla L Gonzalez-Caballero J. Assessment instruments in mental health: description and metric properties. 3rd ed. In: Thornicroft G Tansella M editors. *Mental Health Outcome Measures.* London: The Royal College of Psychiatrists (2010). p. 28–62.

[28] FDA. *Guidance for Industry: Patient-Reported Outcome Measures: Use in Medical Product Development to Support Labeling Claims* (2009). Available online at: www.fda.gov/downloads/Drugs/GuidanceComplianceRegulatoryInformation/Guidances/UCM193282.pdf 10.1186/1477-7525-4-79 PMC1629006 17034633

[29] Moreno-Küstner B Fábrega-Ruz J Gonzalez-Caballero J Reyes-Martin S Ochoa S Romero-Lopez-Alberca C Patient-reported impact of symptoms in schizophrenia scale (PRISS): development and validation. *Acta Psychiatr Scand.* (2022) 145:640–55. 10.1111/acps.13417 35188673

[30] Piotrkowska R Mȩdrzycka-Da̧browska W Jarzynkowski P Ślusarz R. Nicotine dependence and the level of motivation for ceasing smoking in the case of patients undergoing vascular surgeries versus the optimisation of perioperative care-pilot survey. *Int J Environ Res Public Health.* (2022) 19:10393. 10.3390/ijerph191610393 36012032 PMC9408470

[31] Mahmoudi S Rabbani Zadeh M. Development and validation of the rhinoplasty outcomes evaluation (ROE) questionnaire: an analytical study. *World J Plast Surg.* (2022) 11:68–74. 10.52547/wjps.11.2.68 36117897 PMC9446128

[32] Miao Y Wu X Xue X Ma X Yang L Zeng X Morin, the PPARγ agonist, inhibits Th17 differentiation by limiting fatty acid synthesis in collagen-induced arthritis. *Cell Biol Toxicol.* (2022). 10.1007/s10565-022-09769-3 [Epub ahead of print]. 36121554

[33] Russell DW. UCLA Loneliness Scale (Version 3): reliability, validity, and factor structure. *J Pers Assess.* (1996) 66:20–40. 10.1207/s15327752jpa6601_2 8576833

[34] Keyworth C Epton T Goldthorpe J Calam R Armitage C. Acceptability, reliability, and validity of a brief measure of capabilities, opportunities, and motivations (”COM-B”). *Br J Health Psychol.* (2020) 25:474–501. 10.1111/bjhp.12417 32314500

[35] Legge SE Cardno AG Allardyce J Dennison C Hubbard L Pardiñas AF Associations between schizophrenia polygenic liability, symptom dimensions, and cognitive ability in schizophrenia. *JAMA Psychiatry*. (2021) 78:1143–51. 10.1001/jamapsychiatry.2021.1961 34347035 PMC8340009

[36] Enomoto K Adachi T Mibu A Tanaka K Fukui S Nakanishi M Validation of the Japanese version of the patterns of activity measure-pain in individuals with chronic pain. *Biopsychosoc Med.* (2022) 16:19. 10.1186/s13030-022-00248-z 36057611 PMC9441030

[37] Jalali-Farahani S Amiri P Zarani F Zayeri F Azizi F. Development and validation of the body image scale for youth (BISY). *J Eat Disord*. (2022) 10:136. 10.1186/s40337-022-00657-z 36068589 PMC9450403

[38] Manners J Appleton SL Reynolds AC Melaku YA Gill TK Lovato N The Good Sleeper Scale-15 items: A questionnaire for the standardised assessment of good sleepers. *J Sleep Res*. (2022) 32:e13717. 10.1111/jsr.1371 36065002

[39] Mutiso V Musyimi C Tele A Alietsi R Andeso P Ndetei D. Edinburgh Postnatal Depression Scale (EPDS) for screening for depression in the first year post delivery in a low-resourced rural setting in Kenya. *Transcult Psychiatry.* (2022). 10.1177/13634615211043764 [Epub ahead of print]. 34986050

[40] Emsley R Rabinowitz J Torreman M. The factor structure for the Positive and Negative Syndrome Scale (PANSS) in recent-onset psychosis. *Schizophr Res.* (2003) 61:47–57. 10.1016/s0920-9964(02)00302-x 12648735

[41] Kaliuzhna M Kirschner M Carruzzo F Hartmann-Riemer M Bischof M Seifritz E Clinical, behavioural and neural validation of the PANSS amotivation factor. *Schizophr Res.* (2020) 220:38–45. 10.1016/j.schres.2020.04.018 32349887

[42] Wolff A Dresselhuis A Hejazi S Dixon D Gibson D Howard A Healthcare provider characteristics that influence the implementation of individual-level patient-centered outcome measure (PROM) and patient-reported experience measure (PREM) data across practice settings: a protocol for a mixed methods systematic review with a narrative synthesis. *Syst Rev.* (2021) 10:169. 10.1186/s13643-021-01725-2 34108024 PMC8188663

[43] Kendrick T El-Gohary M Stuart B Gilbody S Churchill R Aiken L Routine use of patient reported outcome measures (PROMs) for improving treatment of common mental health disorders in adults. *Cochrane Database Syst Rev.* (2016) 7:CD011119. 10.1002/14651858.CD011119.pub2 27409972 PMC6472430

[44] Buck B Gagen E Halverson T Nagendra A Ludwig K Fortney JC. A systematic search and critical review of studies evaluating psychometric properties of patient-reported outcome measures for schizophrenia. *J Psychiatr Res.* (2022) 147:13–23. 10.1016/j.jpsychires.2021.12.053 35007807 PMC8882143

[45] Bull C Teede H Carrandi L Rigney A Cusack S Callander E. Evaluating the development, woman-centricity and psychometric properties of maternity patient-reported outcome measures (PROMs) and patient-reported experience measures (PREMs): a systematic review protocol. *BMJ Open.* (2022) 12:e058952. 10.1136/bmjopen-2021-058952 35144957 PMC8845328

